# Severe renal haemosiderosis in a patient with untreated paroxysmal nocturnal haemoglobinuria: a case report

**DOI:** 10.1186/s12882-025-04343-5

**Published:** 2025-08-05

**Authors:** Zhong Zhen Goh, Kenny Tang, Katrina Chau, Seethalakshmi Viswanathan

**Affiliations:** 1 Tissue Pathology & Diagnostic Oncology, Institute of Clinical Pathology and Medical Research, Westmead Hospital, Westmead, NSW Australia; 2 Department of Haematology, Blacktown Hospital, Blacktown, NSW Australia; 3 Department of Renal Medicine, Blacktown Hospital, Blacktown, NSW Australia; 4 Westmead Clinical School, University of Sydney, Sydney, NSW Australia; 5 School of Medicine, Western Sydney University, Sydney, NSW Australia

**Keywords:** Renal haemosiderosis, Haemosiderin, Paroxysmal nocturnal haemoglobinuria, Aplastic anaemia

## Abstract

**Background:**

Paroxysmal nocturnal haemoglobinuria (PNH) is a life-threatening disease in which intravascular haemolysis of the red blood cells frequently manifests with chronic haemolysis, anaemia and thrombosis. Renal injury in PNH is associated with chronic haemosiderosis and/or microvascular thrombosis. Herein, we describe a case of haemolytic crisis and severe renal haemosiderosis in a patient who was previously treated for aplastic anaemia (AA) and later developed a symptomatic PNH clone.

**Case presentation:**

A 74-year-old woman with acquired AA treated with immunosuppressive therapy 8 years ago was admitted to our hospital with severe haemolytic anaemia and acute kidney injury in the setting of *Escherichia coli* sepsis. Peripheral blood flow cytometry demonstrated expansion of the small PNH clone detected at diagnosis with clone size now exceeding 80%. Renal biopsy showed extensive brown pigment deposition in most of the proximal tubules and accompanying severe acute tubular injury. The pigment deposits were confirmed to be haemosiderin on Perls’ Prussian blue stain. Based on these biopsy findings and clinical presentation, she was diagnosed with acute tubular injury secondary to *Escherichia coli* sepsis on a background of chronic kidney disease in part due to chronic intravascular haemolysis associated with untreated PNH. During her admission, she was also found to have large vessel vasculitis and was commenced on high-dose steroids. Her acute haemolysis stabilised after treatment of her sepsis and her renal function also improved. A C5 complement inhibitor was commenced following discharge from hospital.

**Conclusion:**

Our case illustrates the potentially severe renal complications of acute on chronic intravascular haemolysis associated with untreated, clinical PNH arising from a history of treated AA. Close monitoring and early intervention of patients with symptomatic PNH is therefore critical.

## Background

Renal haemosiderosis is a condition where haemosiderin, a form of iron, accumulates in the kidneys, specifically the proximal tubular cell [1]. Various conditions associated with intravascular haemolysis may lead to renal haemosiderosis, with paroxysmal nocturnal haemoglobinuria (PNH) being one of the most common causes [1, 2].

PNH is a clonal haematopoietic stem cell disease that frequently manifests with haemolytic anaemia, bone marrow failure and thrombosis [3]. PNH is characterised by an acquired somatic mutation in the *PIGA* gene, resulting in a deficiency of CD55 and CD59, on red cells, leucocytes and platelets, thereby rendering them susceptible to complement-mediated haemolysis [4]. Prolonged intravascular haemolysis may uncommonly lead to renal tubular damage from microvascular thrombosis and accumulation of iron deposits [5]. PNH is closely linked with aplastic anaemia (AA), with 40–60% of patients with AA harbouring a small PNH clone [6]. Patients may develop symptomatic PNH following treatment of AA due to PNH clonal expansion [7]. 

We describe a case of haemolytic crisis and renal haemosiderosis in a patient with clinical PNH following treatment of previously diagnosed AA.

### Case presentation

A 74-year-old woman was admitted to our hospital with malaise and fevers in April 2024. She was previously diagnosed with acquired AA associated with a small PNH clone in 2016 and subsequently treated with Anti-Thymocyte Globulin (ATG) and cyclosporin. She achieved a complete response but developed chronic haemolysis in conjunction with a large PNH clone over subsequent years. In May 2023, she was lost to follow-up before anti-complement therapy could be commenced. Her other medical history included hypertension and type 2 diabetes mellitus.

On admission, her blood pressure was 85/47 mmHg, heart rate was 94 beats per minute, and temperature was 38.2 °C. Physical examination showed jaundice with scleral icterus without abdominal tenderness or hepatosplenomegaly. Laboratory tests revealed severe haemolytic anaemia, renal dysfunction and mild liver dysfunction as outlined in Table 1. Her serum creatinine increased to 187µmol/L from a baseline of 110 µmol/L(eGFR = 43 ml/min/1.73m^2^) recorded in 2023. Urinalysis revealed haematuria (> 100 × 10^6^ RBC/L) but no red cell casts. Her urine albumin: creatinine ratio was 128 mg/mmol. She was started on intravenous antibiotics and received inotropic support for septic shock. She had *Escherichia coli* on urine and blood cultures.

**Table 1:** Laboratory data on admission

	Value on admission	Normal range
**Haematology**		
Haemoglobin	69 g/L	115–165 g/L
Mean corpuscular volume (MCV)	106 fL	82–98 fL
Haematocrit (Hct)	0.2 L/L	0.36–0.44 L/L
White cell count (WCC)	7.7 × 10^9^/L	3.9–11.1 × 10^9^/L
Platelets	92 × 10^9^/L	150–400 × 10^9^/L
Absolute reticulocytes	144 × 10^9^/L	50–100 × 10^9^/L
Reticulocytes %	7.2	0.5–2.5
Haptoglobin	< 0.1 g/L	0.3–2.0 g/L
Direct Antiglobulin Test (DAT)	Negative	-
**Biochemistry**		
Sodium	132 mmol/L	135–145 mmol/L
Potassium	4.3 mmol/L	3.5–5.2 mmol/L
Bicarbonate	19 mmol/L	22–32 mmol/L
Urea	12.2 mmol/L	4–9 mmol/L
Creatinine	187 µmol/L	45–90 µmol/L
Estimated glomerular filtration rate (eGFR)	22 mL/min/1.73m^2^	≥ 90 mL/min/1.73m^2^
Lactate dehydrogenase level (LDH)	2231 U/L	120–250 U/L
C reactive protein (CRP)	267 mg/L	≤ 4 mg/L
**Liver function tests**		
Alanine transaminase (ALT)	44 U/L	10–35 U/L
Aspartate aminotransferase (AST)	191 U/L	10–35 U/L
Gamma-glutamyl transferase (GGT)	65 U/L	5–35 U/L
Alkaline phosphatase (ALP)	107 U/L	30–110 U/L
Total bilirubin	42 µmol/L	≤ 20 µmol/L
**Iron studies**		
Iron levels	4.3 µmol/L	8–30 µmol/L
Transferrin	1.7 g/L	1.8–3.5 g/L
Transferrin saturation	10%	15–45%
Ferritin	304 µg/L	30–300 µg/L

Considering her haemolytic anaemia and previously known PNH clone, PNH flow cytometry was repeated and showed a PNH clone in over 80% of the neutrophil and monocyte population (Table 2).

**Table 2:** Flow-cytometry

Red blood cells (CD235a+)
Partial CD59 GPI deficiency (Type II): 0.07% of total red blood cells
Complete CD59 GPI deficiency (Type III): 3.38% of total red blood cells
GPI linked CD59 deficient population DETECTED in 3.44% (Type II plus Type III) of total Red Blood Cells
GPI linked FLAER/CD59 deficient population DETECTED in 82.04% of total Neutrophils
GPI linked FLAER/CD59 deficient population DETECTED in 83.65% of total Monocytes
A significant GPI deficient population was DETECTED in Neutrophils (CD33^+^CD45^+^), Monocytes (CD33^++^CD45^++^) and/or Red Blood Cells (CD235a^+^)

The patient’s haemolytic crisis stabilised with treatment of her sepsis and with red cell transfusions. However, her renal function continued to deteriorate with serum creatinine rising to 340 µmol/L on day 32 of admission. A provisional diagnosis of acute tubular necrosis was initially considered; however, one would expect renal function to plateau and not to continue declining. Therefore, a renal biopsy was performed on Day 33 of admission to clarify the cause of renal dysfunction, with differential diagnoses including interstitial nephritis and renal vasculitis.

### Kidney biopsy findings

**Fig. 1:**
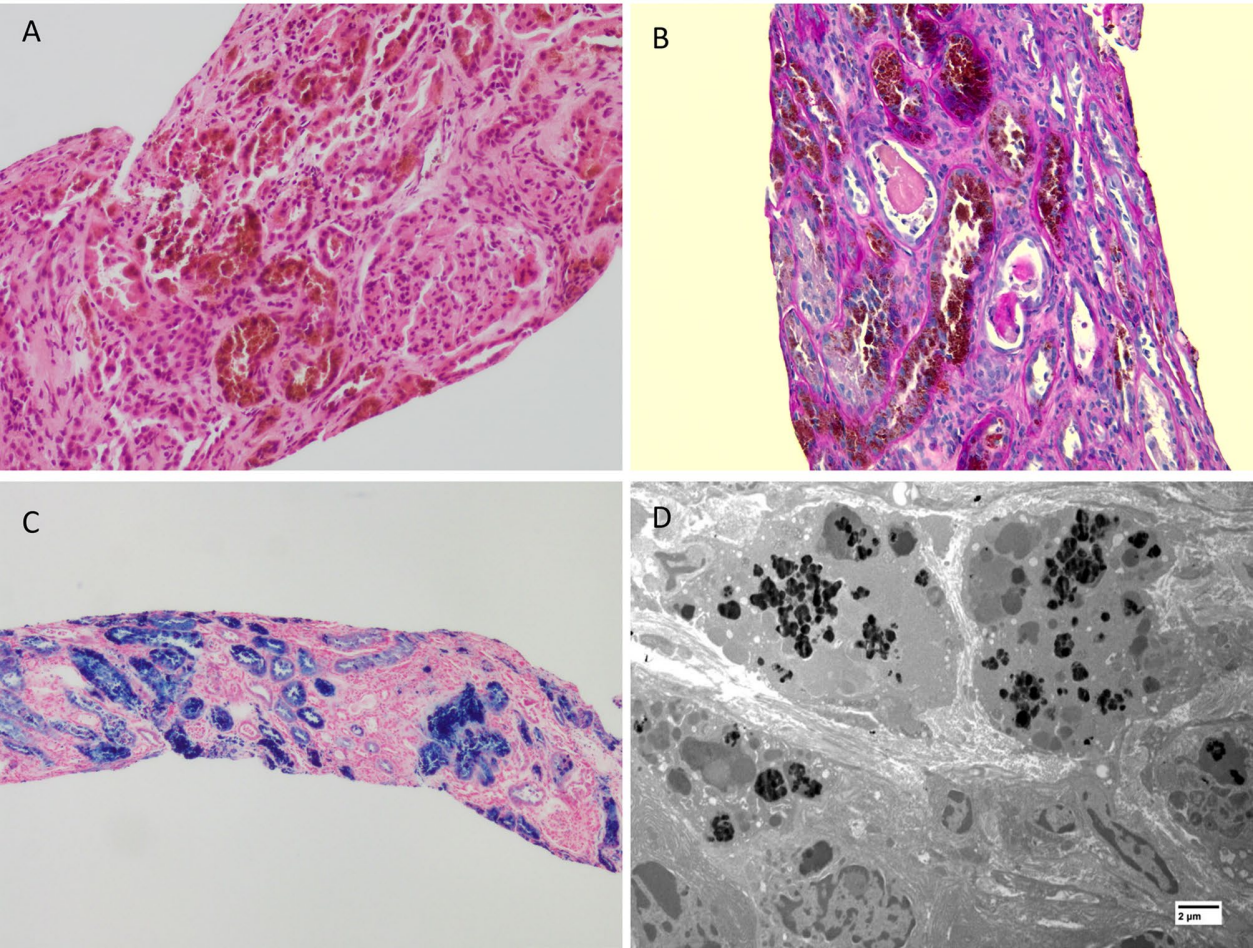
(**A**) Section from the renal biopsy showing brown pigment deposition in many of the proximal tubules within cytoplasm of the tubular epithelial cells. Please note marked background scarring with interstitial fibrosis and tubular atrophy. The glomeruli showed ischemic changes. (H&E X 400) (**B**) PAS stain showing coarse brown granular pigment within proximal tubular epithelial cells amidst scarring in the background (PAS X 400) (**C**) Prussian blue stain confirming the brown pigment to be haemosiderin. Please note the heavy dense staining within many of the tubules indicating the severity of haemosiderin deposition indicative of the long-standing underlying haemolysis (Prussian blue X 200). (**D**) Electron microscopic examination showing electron dense granular deposits within proximal tubular cell cytoplasm (uranyl acetate and lead citrate)

The kidney biopsy contained 5 glomeruli, 1 of which was sclerosed, while the rest were viable. The glomeruli showed ischaemic changes with tuft shrinkage but were otherwise normocellular and unremarkable. The haematoxylin and eosin (H&E) staining showed variable haemosiderin deposition with most proximal tubules showing extensive brown pigment deposition and accompanying severe acute tubular injury (Fig. 1A and B). The pigment deposits were confirmed to be haemosiderin on the Perls’ Prussian blue stain (Fig. 1C). There were severe background changes with interstitial fibrosis and tubular atrophy with moderate to dense active chronic inflammation in the scarred parenchyma. The arteries showed mild intimal fibrosis. The degree of scarring appeared disproportionate to the vascular changes, possibly secondary to the tubulo-interstitial injury due to the haemosiderin deposition. There was no evidence of vasculitis on the biopsy and there were no specific changes related to diabetes or hypertension. A Congo red stain was negative for amyloid. Immunofluorescence microscopy performed with FITC-conjugated anti- IgA, IgM, IgG, C3, C1q, kappa and lambda light chains was negative. Electron microscopy revealed dense amorphous deposits within the cytoplasm of proximal tubular epithelial cells consistent with the haemosiderin deposits seen by light microscopic examination (Fig. 1D). The glomeruli showed non-specific chronic changes and showed no electron dense deposits. Based on these biopsy findings and clinical presentation, a diagnosis of acute tubular necrosis from *Escherichia coli* sepsis on a background of chronic kidney disease due to chronic intravascular haemolysis from untreated PNH was made, with possible pre-existing contribution of hypertension and diabetes.

### Clinical course

During our patient’s hospital admission, she developed multiple cerebral cortical venous thromboses due to PNH. She also had recurrent *Escherichia coli* urosepsis despite an initial treatment response. This resulted in an exacerbation of her chronic haemolysis. Antimicrobial therapy was broadened, however, she continued to have fevers and a markedly raised C-reactive protein. As such, our patient had a positive emission tomography scan which revealed thoracic aortitis. Based on this finding, persistent fevers and raised inflammatory markers in the absence of a new infective focus, she was diagnosed with giant cell arteritis (GCA). The patient was commenced on high-dose corticosteroids and her fevers rapidly resolved while her inflammatory markers improved. Her haemoglobin and renal function also began to recover with serum creatinine plateauing at 160–170 µmol/L (eGFR = 25–27 ml/min/1.73m^2^). She was commenced on ravulizumab, a C5 complement inhibitor, following discharge from hospital and her haemolysis and renal function remain stable 6 months post discharge. The administration of ravulizumab was delayed due to concerns of recurrent sepsis in the setting of concurrent high dose steroids for GCA.

**Fig. 2:**
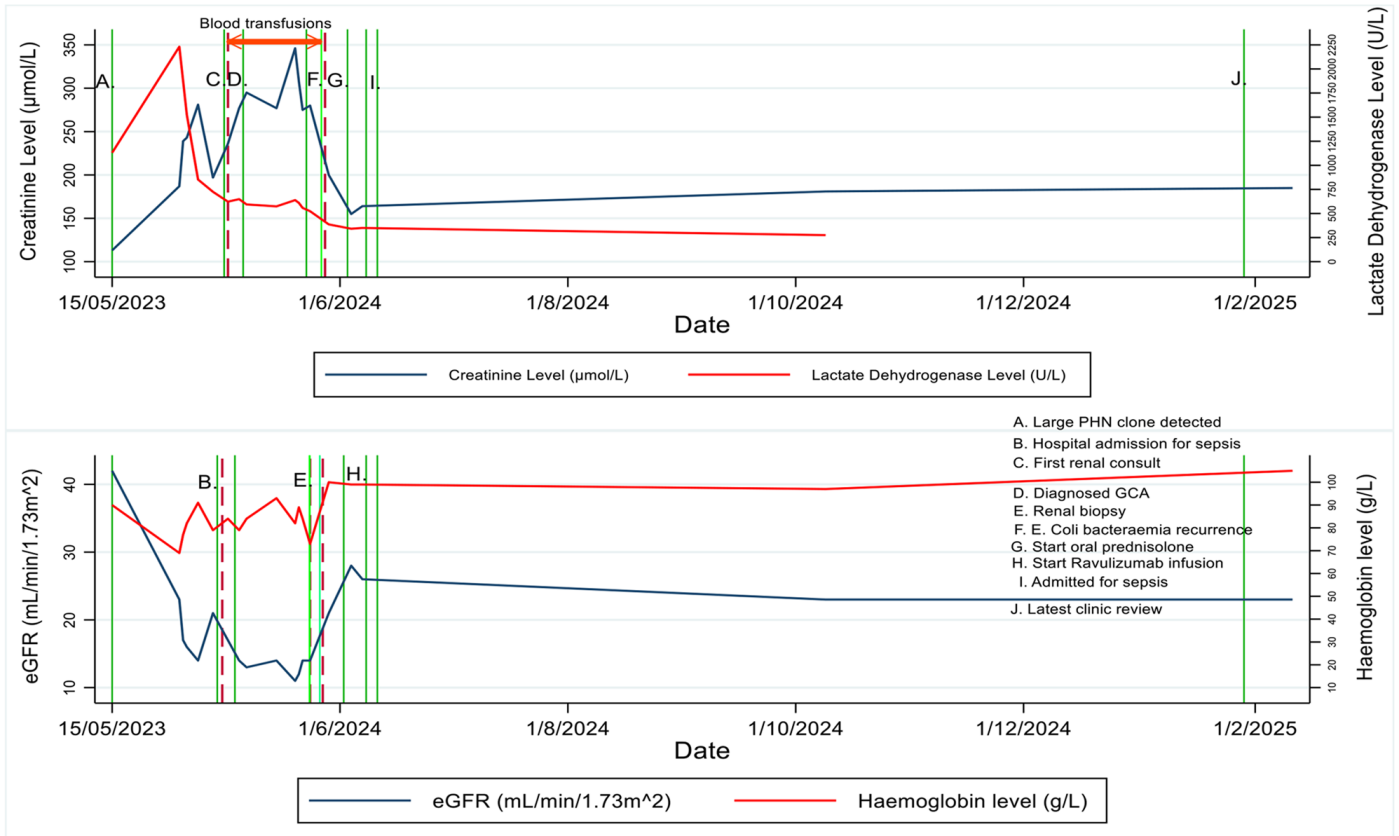
Clinical course – laboratory marker level trends and key clinical events

A detailed clinical course, including the trend of her creatinine, haemoglobin and lactate dehydrogenase levels with key clinical events including transfusion, is outlined in Fig. 2.

## Discussion

Renal haemosiderosis is rare. In most cases, it is only an incidental finding during post-mortem examination of patients with intravascular haemolysis [8]. However, there are also reports of chronic kidney disease resulting from haemosiderin deposition. During intravascular haemolysis, dimeric haemoglobin binds with haptoglobin in the plasma. The haemoglobin-haptoglobin complex is degraded by reticuloendothelial cells. If there is prolonged or massive haemolysis, plasma haptoglobin becomes saturated, free dimeric haemoglobin is allowed to be filtered through the glomeruli and reabsorbed by the proximal tubular cells [9]. In the tubular cells, haemoglobin is catabolised with the release of iron in the form of haemosiderin [10]. There are three proposed mechanisms by which haemosiderin causes kidney injury, including direct cytotoxicity, decreased renal perfusion from depletion of nitric oxide, and cast nephropathy when casts are formed via the interaction of haemosiderin with Tamm-Horsfall protein. In our case, haemosiderosis may be incidental, but given the trajectory of renal function during the clinical course it is a likely contributor to the patient’s chronic kidney disease with the first two mechanisms of haemosiderin injury being possible factors [11]. 

Histologically, renal haemosiderosis manifests as golden/brown pigment deposition in the proximal tubular cells, which stains strongly for Perls’ Prussian blue. There may be the presence of pink-orange pigment casts, however, this was not seen in our case. Acute tubular injury may vary from loss of brush border, cytoplasmic vacuolation, and cellular swelling to extensive necrosis of tubular cells [12]. 

Iron deficiency is commonly seen in PNH and affects 76% of patients with classical (haemolytic) PNH [13]. Iron deficiency may exacerbate the underlying anaemia of PNH through decreased erythropoiesis, which in turn, may lead to increased fatigue and decreased quality of life [14]. In our patient’s case, the haematological profile (ferritin well above > 100 µg/L, mildly reduced transferrin saturation), most likely reflects functional rather than true iron deficiency. That is, total body iron stores are replete but cannot be mobilised for erythropoiesis [15]. 

The exact incidence of renal haemosiderosis in patients with PNH is unknown, but there are case reports of this renal complication [11, 12]. The majority of patients do not have significant renal dysfunction as a result of PNH, with a study by Hillmen et al. showing only 5.1% of their study population having stage 4 or 5 chronic kidney disease [16]. There is conflicting evidence in the literature regarding whether haemosiderosis contributes to renal dysfunction or is an innocent bystander [2, 17]. Herein, we report this rare complication of untreated PNH, where the patient progressed to stage 4 chronic kidney disease, proven on biopsy to be, at least in part, due to haemosiderosis. There was no strong evidence of any other cause for chronic renal impairment, such as diabetic or hypertensive kidney disease, although non-specific findings such as tuft shrinkage and interstitial fibrosis may have been due to these co-morbidities. The failure of her renal function to return to baseline may have also been due to residual damage following acute tubular necrosis. It is likely that in some contexts, such as pre-existing chronic kidney disease that haemosiderosis resulting from prolonged intravascular haemolysis at least accelerates or exacerbates kidney damage.

Our patient’s renal function and anaemia improved following high dose corticosteroid therapy. Whilst GCA does not cause renal dysfunction, the use of corticosteroids may have reduced the effect of interstitial inflammation, which was evident on the renal biopsy. In addition, while corticosteroids may not have directly improved the anaemia caused by chronic haemolysis from PNH, they may have mitigated its severity by addressing the anaemia of inflammation associated with GCA.

The association between PNH and GCA is rare and to the best of our knowledge, only one other case has been previously reported [18]. The exact relationship between PNH and GCA is unclear, but the authors of Tsuyuoka et al. postulated immunological stimulation as a possible common mechanism [18]. From a pathophysiological perspective, PNH and GCA are very different. PNH is a complement-mediated process that causes predominantly venous thromboses [4], while GCA is a T-cell mediated process resulting in arterial thromboses [19]. The clinical implication of this association highlights the importance of meticulously assessing all potential triggers of haemolytic crises and to treat them accordingly to prevent deterioration in renal function related to heightened levels of haemolysis.

## Conclusion

Our case illustrates the potentially severe renal complications of acute on chronic intravascular haemolysis associated with untreated, clinical PNH arising from a previously treated AA. It also highlights the importance of close monitoring of patients following treatment of acquired AA for expansion of an underlying PNH clone, as well as careful screening for all potential triggers of haemolytic crises, as demonstrated by the rare but significant association between PNH and GCA in our patient, so that early intervention can be implemented and the sequelae of persistent, chronic haemolysis and renal haemosiderosis can be mitigated.

## Data Availability

No datasets were generated or analysed during the current study.

## References

[1] Gaut JP, Liapis H. Acute kidney injury pathology and pathophysiology: a retrospective review. Clin Kidney J. 2021;14:526–36. 10.1093/ckj/sfaa142 PMC7886540 33623675

[2] Kokoris SI, Gavriilaki E, Miari A, Travlou Α, Kyriakou E, Anagnostopoulos A, et al. Renal involvement in paroxysmal nocturnal hemoglobinuria: an update on clinical features, pathophysiology and treatment. Hematology. 2018;23:558–66. 29486674 10.1080/10245332.2018.1444563

[3] Parker CJ. Historical aspects of paroxysmal nocturnal haemoglobinuria: ‘defining the disease’. Br J Haematol. 2002;117:3–22. 11918528 10.1046/j.1365-2141.2002.03374.x

[4] Brodsky RA. Paroxysmal nocturnal hemoglobinuria. Blood. 2014;124:2804–11. 10.1182/blood-2014-02-522128. 25237200 10.1182/blood-2014-02-522128 PMC4215311

[5] Shah YB, Priore SF, Li Y, Tang CN, Nicholas P, Kurre P, et al. The predictive value of PNH clones, 6p CN-LOH, and clonal TCR gene rearrangement for aplastic anemia diagnosis. Blood Adv. 2021;5:3216–26. 34427585 10.1182/bloodadvances.2021004201 PMC8405198

[6] Sugimori C, Chuhjo T, Feng X. Minor population of CD55-CD59-blood cells predicts response to immunosuppressive therapy and prognosis in patients with aplastic anemia. Blood. 2006;107:1308–15. 16179371 10.1182/blood-2005-06-2485

[7] Griffin M, Kulasekararaj A, Gandhi S, Munir T, Richards S, Arnold L, Benson-Quarm N, Copeland N, Duggins I, Riley K, Hillmen P, Marsh J, Hill A. Concurrent treatment of aplastic anemia/paroxysmal nocturnal hemoglobinuria syndrome with immunosuppressive therapy and eculizumab: a UK experience. Haematologica. 2018;103:e345–7. 29545341 10.3324/haematol.2017.183046 PMC6068026

[8] Leonardi P, Ruol A. Renal haemosiderosis in the haemolytic anemias: diagnosis by means of needle biopsy. Blood. 1960;16:1029–38. 14415802

[9] Lee IH, Kang GW, Kim CY, Lee SJ, Kim MK, Ahn DJ. Renal haemosiderosis secondary to intravascular haemolysis after mitral valve repair. Med (Baltim). 2020;99:e18798. 10.1097/MD.0000000000018798 PMC7220361 32011483

[10] Taberner K, House AA, Haig A, Hsia CC. A case of renal Iron overload associated with cold agglutinin disease successfully managed by rituximab. Clin Hematol Int. 2023;5:62–6. 38817958 10.46989/001c.91478 PMC10757762

[11] Imafuku A, Yamamoto G, Takemura K, Hasegawa E, Sawa N, Kawada M, et al. Acute kidney injury by renal haemosiderosis secondary to primary cold agglutinin disease associated with an excessive alcohol intake. Intern Med. 2018;57:3261–5. 29984743 10.2169/internalmedicine.0710-17 PMC6287995

[12] Puri V, Gandhi A, Sharma S. Renal biopsy in paroxysmal nocturnal hemoglobinuria: an insight into the spectrum of morphologic changes. Indian J Nephrol. 2017;27:284–8. 28761230 10.4103/0971-4065.202833 PMC5514824

[13] Peng G et al. Oct. Iron deficiency in patients with paroxysmal nocturnal hemoglobinuria: a cross-sectional survey from a Single institution in China. Medical science monitor: international medical journal of experimental and clinical research vol. 24 7256–7263. 11 2018, 10.12659/MSM.910614 10.12659/MSM.910614 PMC6194753 30306969

[14] Waheed A, et al. Paroxysmal nocturnal hemoglobinuria: review of the patient experience and treatment landscape. Blood Reviews Vol. 2024;64:101158. 10.1016/j.blre.2023.101158. 10.1016/j.blre.2023.101158 38071133

[15] Thomas DW, Hinchliffe RF, Briggs C, Macdougall IC, Littlewood T. Cavill, I. and guideline for the laboratory diagnosis of functional iron deficiency. Br J Haematol. 2013;161:639–48. 10.1111/bjh.12311. 23573815 10.1111/bjh.12311

[16] Hillmen P, Elebute M, Kelly R, Urbano-Ispizua A, Hill A, Rother RP, et al. Long-term effect of the complement inhibitor Eculizumab on kidney function in patients with paroxysmal nocturnal hemoglobinuria. Am J Hematol. 2010;85:553–9. 20658586 10.1002/ajh.21757

[17] Hillmen P, Elebute M, Kelly R, Urbano-Ispizua A, Hill A, Rother RP, et al. High incidence of progression to chronic renal insufficiency in patients with paroxysmal nocturnal hemoglobinuria (PNH). Blood. 2007;110(11):3678. 10.1182/blood.V110.11.3678.3678.

[18] Tsuyuoka R, Takahashi T, Shinoda E, Taniguchi Y, Nishibe K, Takeuchi E, Nakao K. Intestinal perforation in Temporal arteritis associated with paroxysmal nocturnal haemoglobinuria. Intern Med. 1996;35:159–61. 8680107 10.2169/internalmedicine.35.159

[19] Koster MJ, Warrington KJ. Giant cell arteritis: pathogenic mechanisms and new potential therapeutic targets. BMC Rheumatol. 2017;1:2. 10.1186/s41927-017-0004-5. 30886946 10.1186/s41927-017-0004-5 PMC6383596

